# Enhanced Desalination Performance of Capacitive Deionization Using Nanoporous Carbon Derived from ZIF-67 Metal Organic Frameworks and CNTs

**DOI:** 10.3390/nano10112091

**Published:** 2020-10-22

**Authors:** Ngo Minh Phuoc, Euiyeon Jung, Nguyen Anh Thu Tran, Young-Woo Lee, Chung-Yul Yoo, Beom-Goo Kang, Younghyun Cho

**Affiliations:** 1 Department of Energy Systems Engineering, Soonchunhyang University, Asan 31538, Korea; minhphuoc7794@gmail.com (N.M.P.); tnathu76@gmail.com (N.A.T.T.); ywlee@sch.ac.kr (Y.-W.L.); 2 Department of Materials Science and Engineering, University of Pennsylvania, 3231 Walnut Street, Philadelphia, PA 19104, USA; r2k3231@gmail.com; 3 Department of Chemistry, Mokpo National University, Muan-gun, Jeollanam-do 58554, Korea; chungyulyoo@mokpo.ac.kr; 4 Department of Chemical Engineering, Soongsil University, Seoul 06978, Korea

**Keywords:** desalination, capacitive deionization, carbon nanotube, metal organic framework

## Abstract

Capacitive deionization (CDI) based on ion electrosorption has recently emerged as a promising desalination technology due to its low energy consumption and environmental friendliness compared to conventional purification technologies. Carbon-based materials, including activated carbon (AC), carbon aerogel, carbon cloth, and carbon fiber, have been mostly used in CDI electrodes due their high surface area, electrochemical stability, and abundance. However, the low electrical conductivity and non-regular pore shape and size distribution of carbon-based electrodes limits the maximization of the salt removal performance of a CDI desalination system using such electrodes. Metal-organic frameworks (MOFs) are novel porous materials with periodic three-dimensional structures consisting of metal center and organic ligands. MOFs have received substantial attention due to their high surface area, adjustable pore size, periodical unsaturated pores of metal center, and high thermal and chemical stabilities. In this study, we have synthesized ZIF-67 using CNTs as a substrate to fully utilize the unique advantages of both MOF and nanocarbon materials. Such synthesis of ZIF-67 carbon nanostructures was confirmed by TEM, SEM, and XRD. The results showed that the 3D-connected ZIF-67 nanostructures bridging by CNTs were successfully prepared. We applied this nanostructured ZIF-67@CNT to CDI electrodes for desalination. We found that the salt removal performance was significantly enhanced by 88% for 30% ZIF-67@CNTs-included electrodes as compared with pristine AC electrodes. This increase in salt removal behavior was analyzed by electrochemical analysis such as cyclic voltammetry (CV) and electrochemical impedance spectroscopy (EIS) measurements, and the results indicate reduced electrical impedance and enhanced electrode capacitance in the presence of ZIF-67@CNTs.

## 1. Introduction

The demand and consumption of natural resources have substantially increased over the last few decades due to the rapid growth of the global population and industrialization. In particular, the shortage of fresh water has been of one of the critical issues facing humanity. The purification of saline water including seawater and brackish water is the most effective alternative for obtaining fresh water, since most water (more than 97%) on earth is seawater. Capacitive deionization (CDI) based on ion electrosorption is an emerging desalination technology due to its low energy consumption and environmental friendliness compared to conventional purification technologies [1,2,3,4,5,6,7,8,9,10]. When an external electric field is applied to a CDI cell, charged ions such as sodium and chloride ions in the saline water are migrated and electrostatically adsorbed onto the oppositely charged electric double layer (EDL) formed at the interface between the electrode and the electrolyte. When the electrode surfaces are saturated with adsorbed ions, a discharging process involving the application of zero or reverse potential is required to remove adsorbed ions, thus allowing for the regeneration of electrodes. Carbon-based materials including activated carbon (AC), carbon aerogel, carbon cloth, and carbon fiber have been mostly used in CDI electrodes due to their high surface area, electrochemical stability, and abundance. However, the low electrical conductivity and non-regular pore shape and size distribution of carbon-based electrodes limits the maximization of the salt removal performance of a CDI desalination system using such electrodes. Various approaches for realizing enhanced desalination performance have been reported so far. The addition of CNT could improve the desalination performance by formation of a conducting bridge [11]. There have also been efforts to improve desalination capacitance by involving redox active materials including metal oxide, conducting polymer, and hybrid materials [12,13,14,15,16]. Metal-organic frameworks (MOFs) have been widely investigated porous materials with periodic three-dimensional structures consisting of metal center and organic ligands. MOFs have attracted substantial attention due to their high surface area, adjustable pore size, periodical unsaturated pores of metal center, and high thermal and chemical stabilities [17,18,19,20,21,22,23]. Therefore, MOFs have been regarded as ideal materials for various applications including gas separation, catalysis, drug delivery systems, and so on [24]. Furthermore, metal ions can be removed, and the frameworks can be carbonized by post-thermal treatment, while maintaining its unique porous structure. It can produce various hybrid products such as metallic oxide, metal decorated carbonaceous materials, and nanoporous carbon structure [25,26,27]. Various types of MOFs have been investigated as electrodes for energy storage systems such as secondary batteries, supercapacitors, and fuel cells. In particular, zeolitic imidazolate frameworks (ZIFs) involving ZIF-8 and ZIF-67 have been widely utilized due to their excellent physical and chemical characteristics and their highly abundant microporous structures [28,29,30,31,32]. It has been reported that nitrogen-doped carbon materials substantially enhance electrical conductivity and provide better wettability of the electrode surface with electrolyte. However, the conductivity of most untreated MOFs is relatively low, which limits their applications for electrochemical devices. Nanostructured carbon materials, including carbon nanotubes (CNTs) and graphene, have been widely investigated as highly conductive agents, which can significantly enhance the electrical interconnectivity between individual electrode particles, including AC [11,33,34,35,36].

In this study, we have synthesized ZIF-67 using CNTs as a substrate to take full advantage of the unique properties of both MOF and nanocarbon materials. Such synthesis of ZIF-67 carbon nanostructures was confirmed by TEM, SEM, and XRD. The results showed that the 3D-connected ZIF-67 nanostructures bridging by CNTs were successfully prepared. We applied this nanostructured ZIF-67@CNT to CDI electrodes for desalination. We found that the salt removal performance was significantly enhanced by 88% for 30% ZIF-67@CNTs-included electrodes compared with pristine AC electrodes. This increase in salt removal behavior was analyzed by electrochemical analysis such as CV and EIS measurements, and the results indicate reduced electrical impedance and enhanced electrode capacitance in the presence of ZIF-67@CNTs.

## 2. Materials and Methods

### 2.1. Materials

Activated carbon (AC, MSP-20X) was purchased from Kansai Coke & Chemicals Co. (Hyogo, Kobe, Japan). Cationic- and anionic-exchange membranes were obtained from Fujifilm (Type 10, Tilburg, The Netherlands). Deionized water was produced using the Human Power II+ purification system (Human Corporation, Seoul, Korea). Multi-wall carbon nanotubes (CNTs) were obtained from Cheap Tubes Inc. (Grafton, VT, USA). Poly (vinylidene fluoride) (PVDF, Mw ~534,000 g/mol), Polyvinylpyrrolidone (PVP, Mw ~40,000 g/mol), and *N*-Methyl-2-pyrrolidone (NMP) were purchased from Sigma-Aldrich Inc. (St. Louis, MI, USA). Sodium chloride (99.5%) and methanol (99.9%) were purchased from Samchun Chemicals Co. (Seoul, Korea). 2-Methylimidazole (C_4_H_6_N_2_, 99%) and Cobalt (II) nitrate hexahydrate (Co(NO_3_)_2_·6H_2_O, 97.7%) were obtained from Thermo Fisher Scientific Co. (Ward Hill, MA, USA). All the chemicals were of analytical grade and were used as received from the suppliers without further purification.

### 2.2. Synthesis of ZIF-67@ CNT

**Figure 1:**
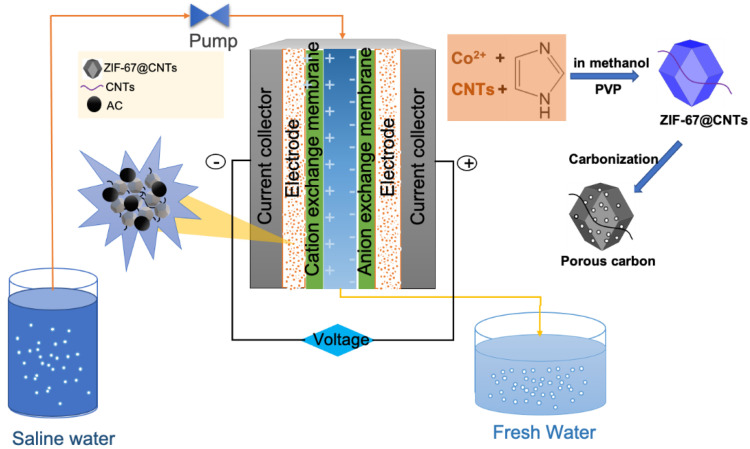
Schematics of capacitive deionization (CDI) desalination system using ZIF-67@CNTs as electrodes.

Cobalt (II) nitrate hexahydrate (Co(NO_3_)_2_·6H_2_O, 0.7270 g) was dissolved in 25 mL of methanol to create solution A, then polyvinylpyrrolidone (PVP, 0.05 g) was added into solution A. In addition, CNTs (50 mg) was dispersed in solution A using an ultrasonicator (NXPC-B2005SB, Seoul, Korea) for 30 min. Solution B was prepared by dissolving 2-methylimidazole (C_4_H_6_N_2_, 2.0520 g) in 25 mL of methanol. Then, solution B was slowly poured into solution A under a continuous stirring condition for 3 h at room temperature (Figure 1). The obtained precipitates were then filtered and washed several times with methanol, and finally dried at 70 °C for 1 h. Synthesized ZIF-67 onto CNTs (ZIF-67@CNTs) were directly calcined in a quartz boat which was placed into a tube furnace for carbon thermal reduction using an electric box furnace (HTF-Q60, Seoul, Korea). The powder was heated with a steadily increasing temperature at a heating rate of 1.2 °C/min for 12 h until reaching 900 °C. At 900 °C, the heating process was maintained for 3 h under high-purity argon gas flow atmosphere. After calcination, the obtained samples were immersed into 2 M HCl solution for 12 h to remove Co species and any impurities. Then, the product was washed several times with DI water to make a neutral environment and dried at 120 °C for 12 h prior to use.

### 2.3. Preparation of CDI Electrode

Each electrode was prepared by pasting a mixture of 90 wt% AC and 10 wt% PVDF dissolved in NMP at a 1:10 ratio. CNTs and ZIF-67@CNTs were added to the electrode mixture and completely mixed using a Thinky mixer (ARM-310, Kidlington, UK) for 10 min. The mixture was subsequently coated onto graphite plates with an area of 2.5 × 4.5 cm by using an automatic thick-film coater (MSK-AFA-II, Zhengzhou, China). Then, the coated electrodes were dried at 70 °C in the drying oven (OF-12GW, Seoul, Korea) for 5 h, and the samples were placed in a vacuum oven (SH-VDO-30NG, Sejong, Korea) for 12 h. The weight and thickness of the prepared electrodes were 0.3 g and 300 µm, respectively, excluding graphite foils.

### 2.4. CDI Operation

As shown in Figure 1, the CDI desalination cell consisted of a symmetrical pair of graphite current collectors. The electrodes including graphite foils were placed onto the current collectors and covered with cationic- and anionic-exchange membranes. Those parts were assembled using a polycarbonate plate and a spacer made of a polyester sheet. The feed solution with a salt concentration of 500 mg/L was continuously pumped into the inlet channel of the CDI cell and drained from the outlet of the CDI cell at a feed rate of 1.8 mL/min using a peristaltic pump (Miniplus 3, Gilson, Inc., Middleton, WI, USA). Desalination was performed by applying the cell potential of 1.2 V for 10 min (charging), and the electrode regeneration was realized by applying the cell potential of −0.3 V for 30 min (discharging) using a potentiostat (ZIVE MP2A, Wonatech, Korea). The conductivity of the effluent stream for ten cycles was measured in real time using a conductivity meter (DS-70 Laqua, Horiba, Japan).

### 2.5. Calculation of Desalination Parameters

The desalination parameters, including the salt adsorption capacity (SAC) and average salt removal rate (ASAR), were calculated using the following Equations:

(1) $$\mathrm{Salt}\;\mathrm{adsorption}\;\mathrm{capacity}\;(\mathrm{SAC})\;(\mathrm{mg}/g)=\frac{\left(C_{i}-C_{e}\right)\times V}{m}$$

(2) $$\mathrm{Average}\;\mathrm{salt}\;\mathrm{adsorption}\;\mathrm{rate}\;(\mathrm{ASAR})\;(\mathrm{mg}/g\;\min)=\frac{SAC}{t}$$

where *C_i_* and *C_e_* are the initial and final salt concentrations, respectively (mg/L), *V* is the volume of the NaCl solution flowing through the cell (L), *m* is the mass of both electrodes without graphite foil (g), and *t* is the charging time (min).

### 2.6. Electrode Characterization

To analyze the specific surface area (SSA), gas sorption measurements (BELSORP max, Microtrac MRB, Osaka, Japan) were conducted by the Brunauer–Emmett–Teller (BET) method, and the pore volume was calculated by the density functional theory (DFT). X-ray diffraction (XRD) patterns of the samples were obtained by MiniFlex600 (Rigaku Corporation, Tokyo, Japan) using CuKα radiation (40 kV, 15 mA). The surface morphology and energy dispersive X-ray spectroscopy were investigated using JEOL 7800F Prime (Akishima, Tokyo, Japan) operating at 15 kV and a transmission electron microscope (JEOL JEM-2100F, Akishima, Tokyo, Japan) operating at 200 kV.

### 2.7. Electrochemical Characterization

Cyclic voltammetry (CV) measurements were performed in the potential range of 0 to 1 V by using a potentiostat (Multi Autolab/M204, Metrohm AG, Herisau, Switzerland) in a three-electrode system consisting of 1 M NaCl electrolyte solution, a counter electrode made of platinum (Pt), a working electrode made of the active materials, and an Ag/AgCl reference electrode. The specific capacitance was calculated using the following Equation:

(3) $$C\;(F/g)=\frac{\int IdV}{2v\Delta Vm}$$

where C is the specific capacitance (F/g), *I* is the response current density (A), *v* is the scan rate (V/s), ∆*V* is the applied voltage window (V), and *m* is the mass of the active materials without graphite foils (g). Electrochemical impedance spectroscopy (EIS) measurements were conducted using ZView software (Wonatech, Seoul, Korea) in the frequency range from 1 kHz to 0.05 Hz in 500 mg/L NaCl solution.

## 3. Results and Discussion

**Figure 2:**
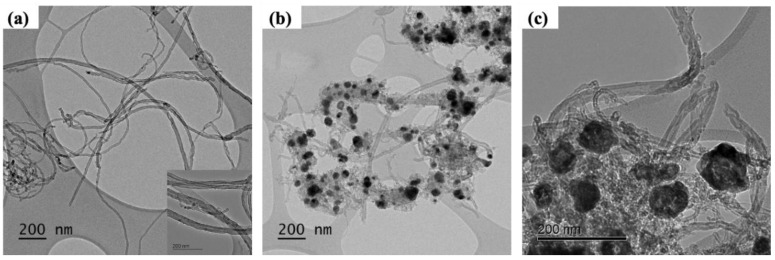
TEM images of (**a**) pristine carbon nanotubes (CNTs), (**b**) low- and (**c**) high-magnification images of ZIF-67 synthesized onto CNTs after carbonization. The inset of (**a**) is a high-magnification image of pristine CNTs.

Figure 2 shows the TEM images of ZIF-67 synthesized onto CNTs. The pristine CNTs have an outer diameter of around 40 nm with a wall thickness of 10 nm, as shown in Figure 2a. Figure 2b,c show that ZIF-67 polyhedron nanoparticles with a size of around 50–100 nm were successfully grown onto CNTs. Furthermore, ZIF-67 grown onto CNTs in this way were additionally carbonized at 900 °C for 12 h to increase the electrical conductivity and the surface area. The ZIF-67 particles were homogeneously and closely distributed onto CNTs even after carbonization.

**Figure 3:**
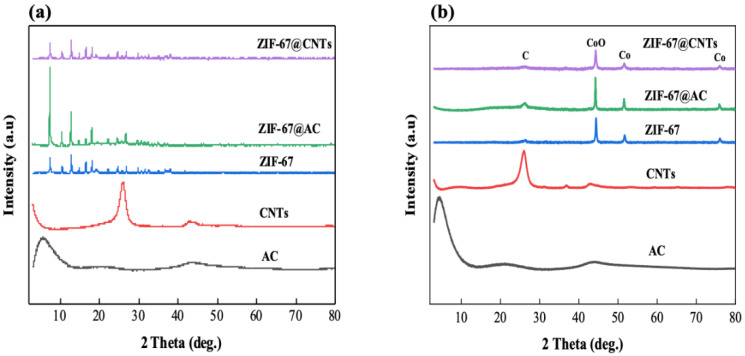
XRD patterns of various samples (**a**) before heating treatment and (**b**) after carbonization at 900 °C.

The crystalline structures of the synthesized ZIF-67 were characterized by X-ray Diffraction (XRD) analysis, as shown in Figure 3. AC and CNTs showed the characteristic peaks at 5.6° and 43.7° (AC) and 26.1° and 42.9° (CNTs) of 2θ originating from the (002) and (100) planes, respectively, of the graphite structure. Freestanding ZIF-67 synthesized without CNT or AC showed the main characteristic crystalline peaks at 7.4°, 12.9°, 16.6°, and 18.2° of 2θ as well as some other minor peaks, which are consistent with the findings of previous studies [37,38]. When ZIF-67 was synthesized onto AC and CNTs, its characteristic peaks were well maintained, as shown in Figure 3a, suggesting that all ZIF-67 are pure with high crystallinity. Following carbonization at 900 °C for 12 h, the characteristic peaks of AC and CNTs were well maintained. By contrast, the ZIF-67 samples, including ZIF-67 onto AC and CNTs, showed structural transformations after carbonization. The main ZIF-67 characteristic peaks, and the long-range ordered crystalline peaks in particular, mostly disappear. At the same time, the broad (002) peak and (111), (200), and (220) peaks were generated. The peaks at 26.2°, 44.3°, 51.6°, and 75.9° of 2θ represent the carbonaceous materials ((002)), Co and CoO ((111), (200), and (220)), respectively [39,40,41]. This indicates the complete conversion of pristine ZIF-67 to nanocarbon structures and the transformation of the ZIF-67 framework during carbonization. The morphological characterizations, including TEM (Figure 2) and XRD (Figure 3), confirm that the uniform ZIF-67 nanoparticles were successfully synthesized onto CNT templates.

**Figure 4:**
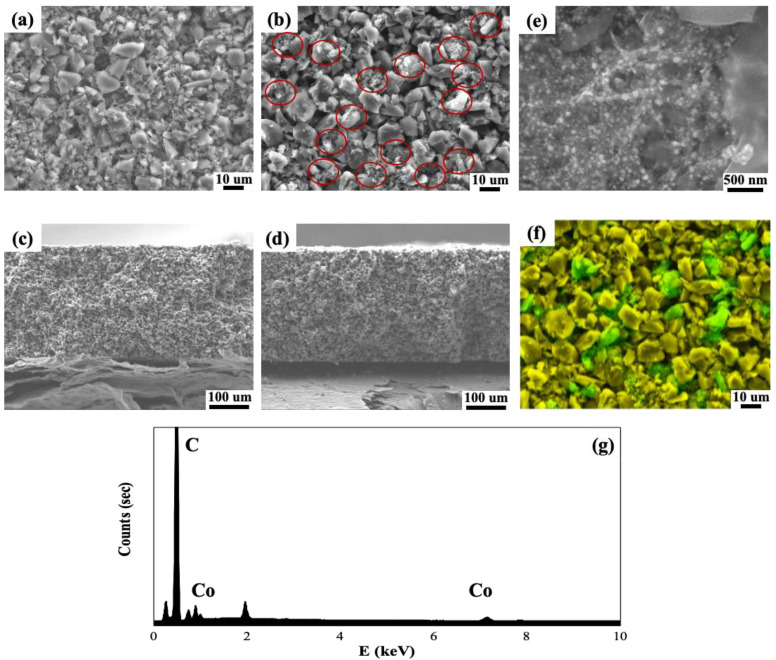
FE-SEM images of CDI electrodes coated onto graphite foil consisting of activated carbon (AC) only (**a**,**c**) and AC with ZIF-67@CNTs (**b**,**e**,**d**). (**a**,**b**,**e**) show top views and (**c**,**d**) show cross-sectional views. (**e**) High-magnification image of AC with ZIF-67@CNTs. (**f**) Corresponding EDS elemental mapping analysis of FE-SEM image (**b**). (**g**) EDS spectrum of AC with ZIF-67@CNTs electrode.

To utilize ZIF-67 synthesized onto CNTs as CDI electrode materials, such samples were included in activated carbon (AC) particles and coated onto graphite foil. For conventional CDI electrodes, AC has mostly been used due to its high surface area, chemical stability, and low cost. To achieve the best performance, binder materials and conducting agents are typically added in the ratio of AC (~80%), binder (~10%), and conducting agents (~10%). However, since one-dimensional CNTs with extremely high electrical conductivity and aspect ratios can act as efficient conducting materials, only 10 wt% binder (PVDF) has been included in the CDI electrode, without any conducting agent materials. Figure 4 shows field emission-scanning electron microscopy (FE-SEM) images (top and cross-sectional views) and Energy dispersive X-ray spectroscopy (EDS) elemental mapping analyses of pristine AC and AC with 10 wt% ZIF-67@CNT electrodes. Compared to pristine AC electrodes (Figure 4a,c), ZIF-67@CNT are uniformly distributed through the whole AC particle, as shown in the red circles in Figure 4b. Therefore, it is confirmed that such ZIF-67@CNT can effectively connect individual AC particles, and can thus act as conducting bridges (Figure 4e). Furthermore, since the ZIF-67@CNT materials, which were relatively small compared to AC particles (~10 μm in average size), were efficiently penetrated in the voids between AC particles, the electrode thickness of the ZIF-67@CNT-included AC electrode was identical to that of the pristine AC electrode (~300 μm, as shown in Figure 4c,d). This indicates that the addition of ZIF-67@CNT into the AC electrode does not affect the physical electrode characteristics, indicating no sacrifice in the volumetric efficiency of the CDI desalination cell, even with the inclusion of additional materials into the typical AC electrode. The EDS elemental analysis results (Figure 4f,g) also confirmed the spatial distribution of the cobalt element originating from ZIF-67 through the electrodes (carbon element in yellow color and cobalt element in green color), which corresponds to the ZIF-67@CNT shown in Figure 4b (red circles).

**Figure 5:**
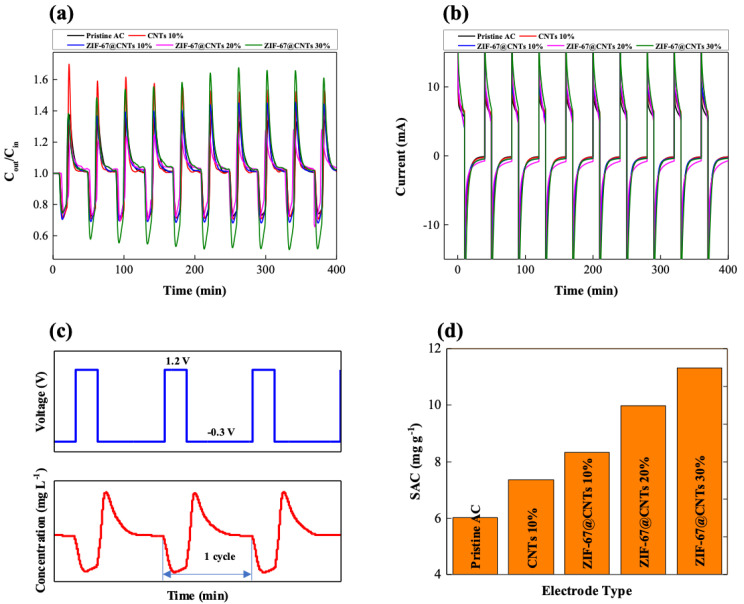
Variation in the (**a**) salt concentration of effluent stream and (**b**) measured current during CDI desalination for 10 cycles. (**c**) Potential difference applied to CDI cell (**top**) and change in effluent concentration magnified from (**a**) (**bottom**). (**d**) Calculated salt adsorption capacity of various CDI electrodes.

**Figure 6:**
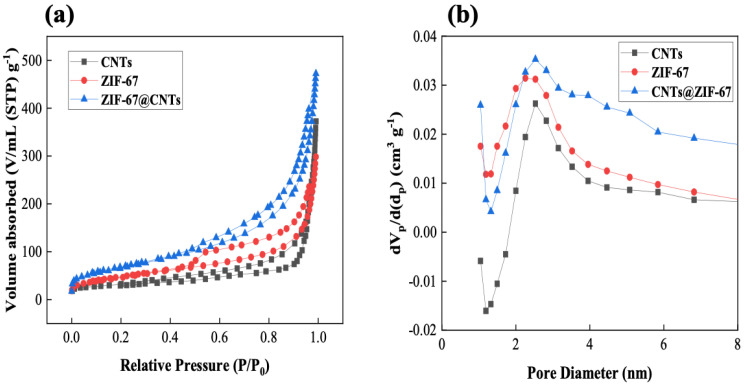
(**a**) Nitrogen absorption/desorption isotherms and (**b**) pore size distributions of the various synthesized materials in this study.

The salt removal performance of the synthesized ZIF-67@CNT-added electrodes in the CDI desalination system have been investigated, and the results are shown in Figure 5. The CDI desalination cell was charged at 1.2 V for 10 min, then subsequently discharged for 30 min, and this process was repeated for 10 cycles as shown in Figure 5c. During the charging process, positively charged sodium and negatively charged chloride ions were electro-adsorbed onto the oppositely charged electric double layer of CDI electrodes through electrostatic interaction. Therefore, the effluent salt concentration dropped immediately at the beginning of the charging process and subsequently increased gradually as the electrode surfaces were fully saturated with adsorbed salt ions, as shown in Figure 5c, because salt ions were no longer adsorbed onto the electrode. During the discharging process at reverse potential (−0.3 V), salt ions adsorbed onto the electrode surface were desorbed due to the repulsive interaction, resulting in an increased salt concentration of effluent stream compared to that of influent stream. This change in salt concentration was repeated throughout the entirety of CDI operation, as shown in Figure 5a and b. The salt adsorption capacity (SAC) of pristine AC representing the salt removal performance of CDI electrodes was 6.01 mg/g. It increased to 7.35 mg/g in the presence of 10 wt% CNT, and further significantly increased up to 8.32, 9.98, and 11.32 mg/g for 10, 20, and 30 wt% ZIF-67@CNT, respectively, which are increases of 38%, 66%, and 88% in SAC compared with the pristine AC electrode. Furthermore, it showed stable long-term operation stability up to 1000 min as shown in Figure S1. To ensure an accurate comparison of the salt removal performance of various CDI electrodes, the electrode thickness was matched to 300 μm. For the conventional preparation of CDI electrodes, carbon black nanoparticles were introduced to form electric bridges between individual AC particles. When CNTs were included in the AC electrodes, they could efficiently act as conducting bridges, resulting in increased salt removal performance (6.01 to 7.35 mg/g in SAC) even without carbon black nanoparticles. N_2_ adsorption and desorption isotherms were analyzed to investigate the surface area and pore size distribution of the various electrode materials, as shown in Figure 6 and Table 1. The specific surface area was calculated by the Brunauer–Emmett–Teller (BET) method, and the pore size distributions were calculated by density functional theory (DFT). All of the samples exhibited N_2_ isotherms similar to type IV. Specifically, for pristine ZIF-67 and ZIF-67@CNTs, they increased at a low relative pressure, which indicates that the micropores were the major vacancy of the porous structures. Furthermore, the hysteresis between adsorption–desorption curves indicates that mesopores also existed in the porous structures. The specific surface area (S_BET_)of CNTs was ~110.7 m^2^/g, which was much lower than that of pristine AC (2395.6 m^2^/g). Therefore, it is believed that the higher salt removal performance in the presence of CNTs mainly originated from the enhanced conductivity rather than the direct ion adsorption onto the CNT surfaces. Furthermore, for ZIF-67@CNTs, SAC increased more significantly as more ZIF-67@CNTs were introduced into the AC electrodes (10 to 30 wt%). The specific surface areas of ZIF-67 and ZIF-67@CNTs were 169.6 and 239.9 m^2^/g, respectively, which were higher than that of pristine CNTs. It has been previously reported that such coexistence of micro- and mesopores of ZIF can provide ion adsorption sites and an ion transport pathway [42]. The N_2_ adsorption and desorption of AC and ZIF-67@CNT without carbonization typically shows a type 1 isotherm, indicating that the micropores are the major vacancies of the structures. The proper pore size distribution of porous electrodes enhances the electrosorption of ions on the electrode materials, even though the specific surface areas of such AC and ZIF-67@CNT without carbonization are higher than that of ZIF-67@CNT after carbonization (Figure S2). Ions can be more readily transported through mesopores and can be adsorbed in micropores. The combination of the enhanced conductivity originating from CNTs and the proper pore size distribution originating from ZIF-67 after carbonization provides substantially more enhanced salt removal performance in CDI desalination (Figure S3).

**Figure 7:**
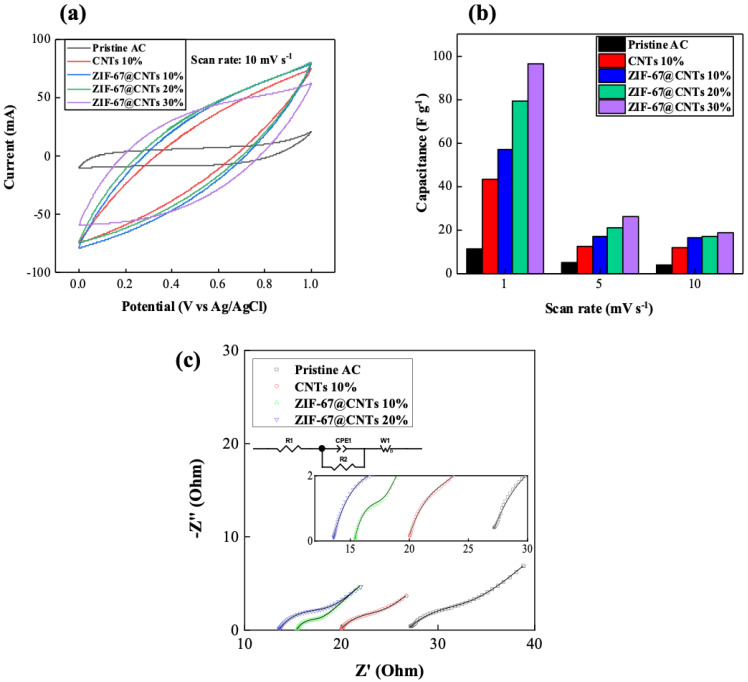
(**a**) Cyclic voltammetry (CV) curves measured for CDI cells at a scan rate of 10 mV/s. (**b**) Change in capacitance calculated from CV measurements at various scan rates. (**c**) Electrochemical impedance spectroscopy (EIS) data (symbols) measured under CDI operation mode with various electrodes and fitting result (line) using corresponding equivalent circuit.

The electrochemical performances of the CDI cell using various synthesized CDI electrodes were investigated by CV as shown in Figure 7. The CV test was performed with a three-electrode system consisting of the counter electrode (Pt), the working electrode (active material), and the reference electrode (Ag/AgCl) in a 1M NaCl electrolyte. Figure 7a shows the CV curves of various electrodes at a scan rate of 10 mV/s, respectively. All the CV plots showed nearly rectangular shapes and no other redox peaks, indicating that sodium and chloride ions were electrosorbed onto the electrical double layer (EDL) generated on the electrode surface rather than the contribution from pseudocapacitive or redox reactions. As pristine CNT was included and the concentration of ZIF-67@CNT was increased (10 to 30%), there was a larger CV closed area. This indicates that the specific capacitance of the CDI electrodes increased. The specific capacitance of the pristine AC electrode was 3.6, and it increased to 11.7 F/g with 10% CNTs. In addition, it gradually increased to 16.4, 16.7, and 18.9 F/g for 10%, 20%, and 30% ZIF-67@CNT-included electrodes, respectively. As the scan rate decreased from 10 to 1 mV/s, the specific capacitance systematically increased in the manner shown in Figure 7b. At the scan rate of 1 mV/s, 30% ZIF-67@CNTs exhibited the highest specific capacitance of 96.6 F/g (Figure S4).

Electrochemical impedance spectroscopy (EIS) measurement was also conducted under the open-circuit voltage condition. Figure 7c shows the Nyquist plots of the impedance spectra of the CDI using various electrodes fitted to the corresponding equivalent circuit. The EIS fitting parameters are summarized in Table S1. For the equivalent circuit of the CDI cell, R1 represents the internal resistance, including the electrolyte and membrane resistances. R2 and CPE1 represent the resistance and constant phase element (CPE), respectively, implying the interfacial adsorption of ions on the electrode surfaces. W1 is the finite-length Warburg impedance representing the ion diffusion from the electrolyte to the electrode pores. For the 10% CNT electrode, R1 decreased from 26.8 Ω (pristine AC) to 19.9 Ω. It further decreased to 15.3 Ω and 13.5 Ω for 10% and 20% ZIF-67@CNT, respectively. The addition of CNT improved the electrical conductivity of AC by forming electrical contacts between AC particles, as reported in a previous study [11], and the addition of ZIF-67@CNT increased the surface area of the electrode by about 87% in comparison with the 10% CNT electrode (See Figure 6c). Therefore, R1 was found to decrease in the order of AC > 10% CNT > 10% ZIF-67@CNT > 20% and ZIF-67@CNT, while R2 decreased from 5.99 Ω (pristine AC) to 3.73 Ω and 1.81 Ω for 10% CNT and 10% ZIF-67@CNT, respectively. This indicates that the ion adsorption reaction at the surface of AC was enhanced by providing electrical contacts between AC particles by CNT and the ion adsorption sites of ZIF67, respectively. However, R2 increased from 1.81 Ω to 4.07 Ω for 10% ZIF-67@CNT and 20% ZIF-67@CNT, respectively, suggesting that the ion adsorption reaction of ZIF-67 might have been hindered due to the increased interface between the ZIF-67 and the AC matrix when ZIF-67@CNT exceeded 10%. The ion diffusion coefficients of CDI electrodes followed the order of 20% ZIF-67@CNT > 10% ZIF-67@CNT > 10% CNT > pristine AC since the diffusion coefficient is inversely proportional to the WO-T and the square of WO-R, and this is consistent with previously reported desalination experiments [43].

## 4. Conclusions

In this study, we have synthesized a ZIF-67 metal organic framework onto CNTs and used them as electrodes in the CDI desalination system. Various characterization results including FE-SEM, TEM, EDS, and XRD confirmed that the ZIF-67 nanoparticles were successfully prepared and homogeneously distributed onto CNTs. The CV measurement results exhibited that the salt removal capacitance significantly increased when ZIF-67@CNTs was added into AC, as compared with those of electrodes composed of only AC or pristine CNT-added AC. Specifically, the salt removal capacitance (SAC) was greatly enhanced from 6.01 (pristine AC) to 11.32 mg/g (30% ZIF-67@CNTs), which represents an 88% increase in salt removal performance. The nitrogen absorption/desorption and EIS experimental results indicated that such enhanced salt removal behavior originated from the combination of the enhanced conductivity from CNTs and proper pore size distribution from ZIF-67, which is much higher compared with the electrodes including only CNTs or ZIF-67. Furthermore, since the salt removal behavior of ZIF-67@CNT results from the capacitive process instead of redox reaction, it showed highly stable long-term stability. We believe that our approach can open a new door for realizing high-performance deionization for the desalination of more highly concentrated brackish water, and could have potential for large-scale desalination modules.

## Figures and Tables

**Table 1:** Specific surface areas and pore volumes of pristine CNTs, ZIF-67, and ZIF-67@CNTs.

Samples	S_BET_ (m^2^/g)	V_total_ (cm^3^/g)
CNTs	110.7	0.55
ZIF-67	169.6	0.45
ZIF-67@CNTs	239.9	0.71

## Supplementary Materials

The following are available online at https://www.mdpi.com/2079-4991/10/11/2091/s1. Figure S1. Variation in the (a) salt concentration of effluent stream and (b) measured current during CDI desalination for 25 cycles using 30 wt% ZIF-67@CNT. Figure S2. (a) Nitrogen absorption/desorption isotherms and (b) pore size distributions of ZIF-67@CNT before and after carbonization (c) Specific surface areas and pore volumes of ZIF-67@CNT before and after carbonization. Figure S3. Variation in the (a) salt concentration of effluent stream and (b) measured current during CDI desalination for 10 cycles using ZIF-67@CNT without carbonization. (c) Comparison on salt removal capacity of various CDI electrodes. Figure S4. (a) CV curves measured for CDI cells with various electrode materials at scan rates of (a) 1 and (b) 5 mV/s, Table S1. EIS fitting parameters for CDI cell using various electrode materials.
