# Supplementary material for: Enhanced Desalination Performance of Capacitive Deionization Using Nanoporous Carbon Derived from ZIF-67 Metal Organic Frameworks and CNTs
Source: Nanomaterials (Basel). 2020 Oct 22;10(11):2091. doi: 10.3390/nano10112091 (PMC7690409; doi:10.3390/nano10112091)
Supplement: Supplementary file 1 [file nanomaterials-10-02091-s001.pdf]

nanomaterials

Supplementary Materials

Enhanced Desalination Performance of Capacitive Deionization Using Nanoporous Carbon Derived from ZIF-67 Metal Organic Frameworks and CNTs

Ngo Minh Phuoc [1,†], Euiyeon Jung [2,†], Nguyen Anh Thu Tran [1], Young-Woo Lee [1], Chung-Yul Yoo [3], Beom-Goo Kang [4,*] and Younghyun Cho [1,*]

[1] Department of Energy Systems Engineering, Soonchunhyang University, Asan 31538, Korea; minhphuoc7794@gmail.com (N.M.P.); tnathu76@gmail.com (N.A.T.T.); ywlee@sch.ac.kr (Y.-W.L.)
[2] Department of Materials Science and Engineering, University of Pennsylvania, 3231 Walnut Street, Philadelphia, PA 19104, USA; r2k3231@gmail.com
[3] Department of Chemistry, Mokpo National University, Muan-gun, Jeollanam-do 58554, Korea; chungyulyoo@mokpo.ac.kr
[4] Department of Chemical Engineering, Soongsil University, Seoul 06978, Korea
* Correspondence: bkang@ssu.ac.kr (B.-G.K.); yhcho@sch.ac.kr (Y.C.); Tel.: +82-2-820-0615 (B.-G.K.); +82-41-530-1720 (Y.C.)
† The first two authors contributed equally to this work.

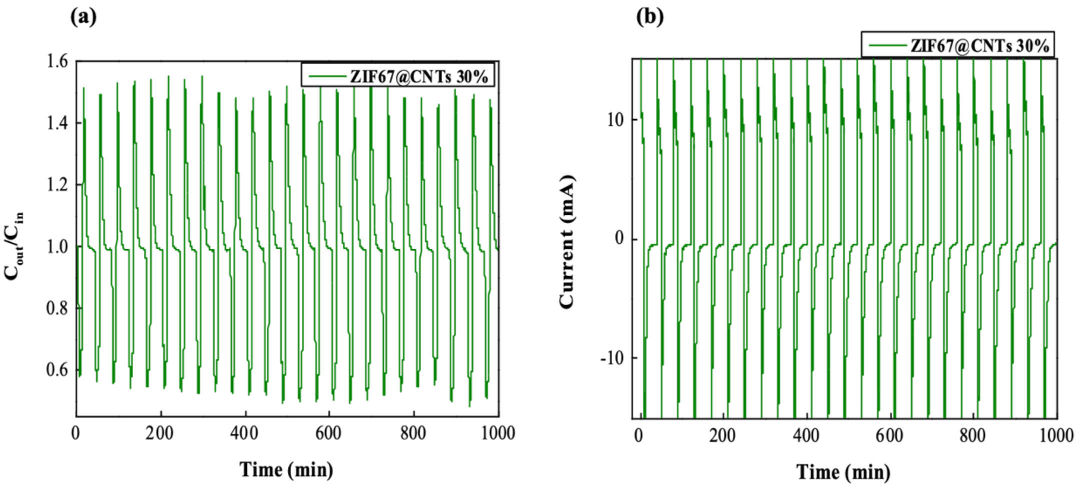

Figure S1. Variation in the (a) salt concentration of effluent stream and (b) measured current during CDI desalination for 25 cycles using 30 wt% ZIF-67@CNT.

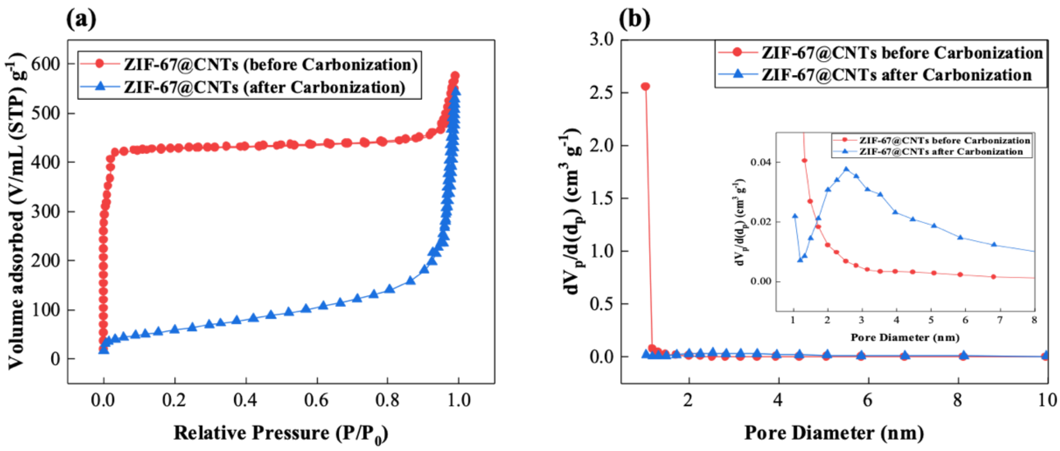

(c)

Samples	S_{BET} (m²/g)	V_{total} (cm³/g)
ZIF-67@CNTs before Carbonization	1662.2	0.88
ZIF-67@CNTs after Carbonization	239.9	0.71

Figure S2. (a) Nitrogen absorption/desorption isotherms and (b) pore size distributions of ZIF-67@CNT before and after carbonization (c) Specific surface areas and pore volumes of ZIF-67@CNT before and after carbonization.

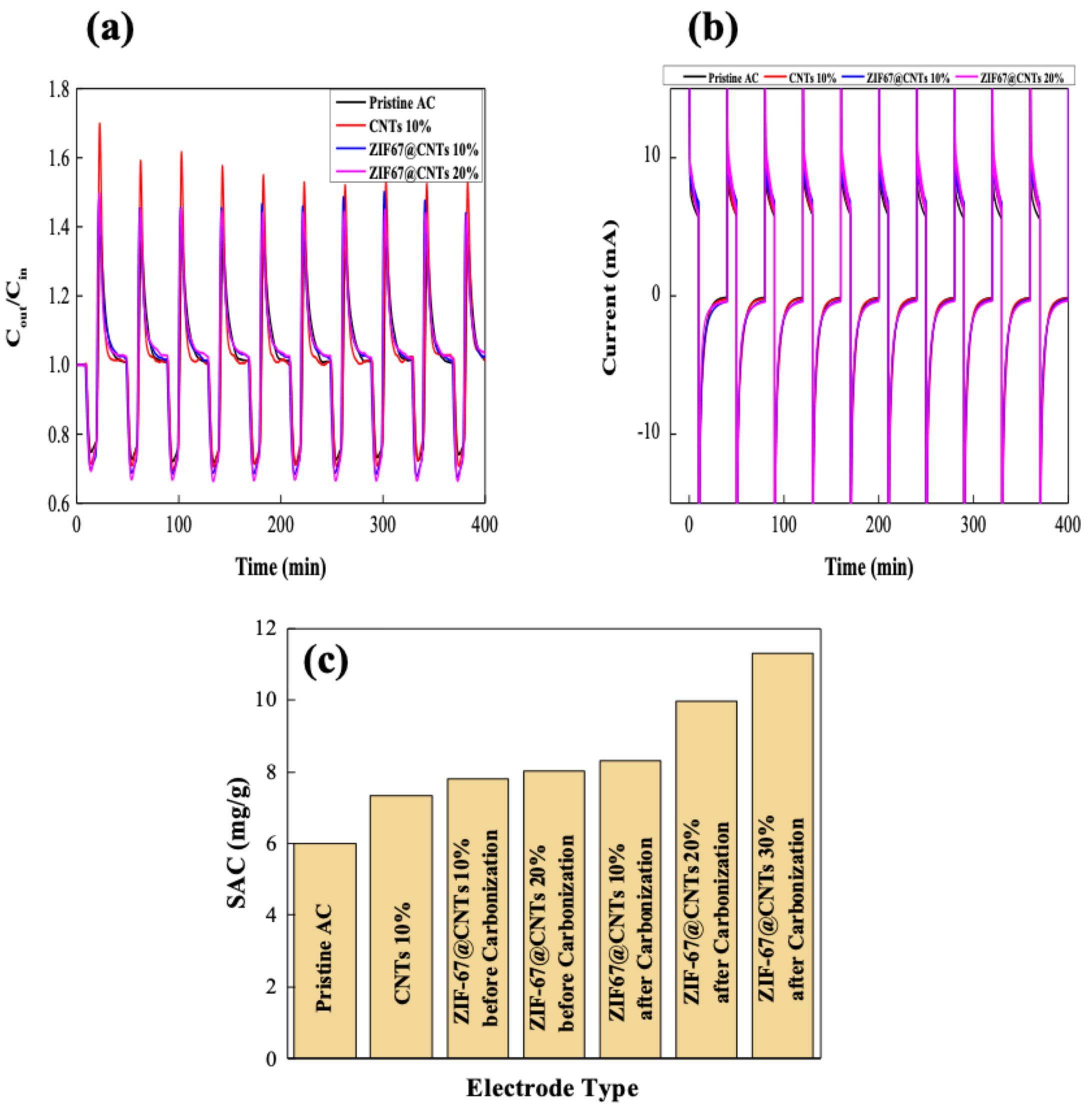

Figure S3. Variation in the (a) salt concentration of effluent stream and (b) measured current during CDI desalination for 10 cycles using ZIF-67@CNT without carbonization. (c) Comparison on salt removal capacity of various CDI electrodes.

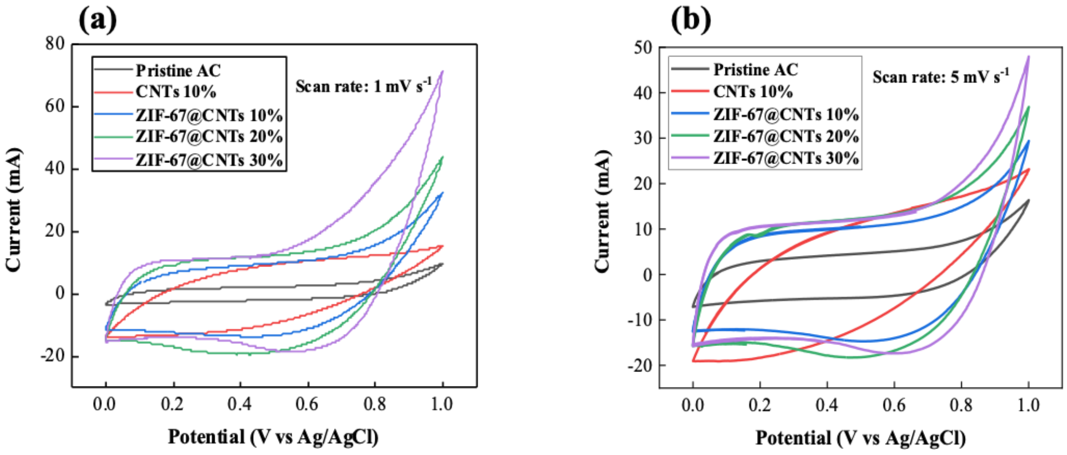

Figure S4. (a) CV curves measured for CDI cells with various electrode materials at scan rates of (a) 1 and (b) 5 mV/s.

Table S1. EIS fitting parameters for CDI cell using various electrode materials.

	AC	CNT 10%	ZIF-67@CNT 10%	ZIF-67@CNT 20%
R1 (Ω)	26.83 (±0.02)	19.89 (±0.01)	15.34 (±0.01)	13.51 (±0.01)
R2 (Ω)	5.99 (±0.01)	3.73 (±0.01)	1.81 (±0.02)	4.07 (±0.02)
CPE1 ($\Omega^{-1}\cdot s^{-n}$)	3.64×10^{-2} ($\pm 8.71 \times 10^{-4}$)	7.17×10^{-2} ($\pm 1.39 \times 10^{-3}$)	3.73×10^{-2} ($\pm 1.20 \times 10^{-3}$)	4.02×10^{-2} ($\pm 3.59 \times 10^{-3}$)
n1	0.55 (±0.01)	0.61 (±0.01)	0.77 (±0.01)	0.70 (±0.01)
WO-R (Ω)	88453 (±592)	3067 (±1022)	290 (±12)	287 (±2)
WO-T (s)	3.06×10^{8} ($\pm 4.23 \times 10^{6}$)	1.33×10^{6} ($\pm 6.06 \times 10^{4}$)	6892 (±143)	6535 (±62)
WO-P	0.5	0.5	0.5	0.5
